# Rapid Fabrication of Anatomically-Shaped Bone Scaffolds Using Indirect 3D Printing and Perfusion Techniques

**DOI:** 10.3390/ijms21010315

**Published:** 2020-01-02

**Authors:** Brian E. Grottkau, Zhixin Hui, Yang Yao, Yonggang Pang

**Affiliations:** The Laboratory for Therapeutic 3D Bioprinting, Department of Orthopaedic Surgery, Massachusetts General Hospital, Harvard Medical School, Boston, MA 02114, USA; zhui@mgh.harvard.edu (Z.H.); 3dbiopr@gmail.com (Y.Y.)

**Keywords:** 3D Printing, perfusion, bone, anatomically-shaped mold, tissue engineering

## Abstract

Fused deposit modeling (FDM) 3D printing technology cannot generate scaffolds with high porosity while maintaining good integrity, anatomical-surface detail, or high surface area-to-volume ratio (S/V). Solvent casting and particulate leaching (SCPL) technique generates scaffolds with high porosity and high S/V. However, it is challenging to generate complex-shaped scaffolds; and solvent, particle and residual water removal are time consuming. Here we report techniques surmounting these problems, successfully generating a highly porous scaffold with the anatomical-shape characteristics of a human femur by polylactic acid polymer (PLA) and PLA-hydroxyapatite (HA) casting and salt leaching. The mold is water soluble and is easily removable. By perfusing with ethanol, water, and dry air sequentially, the solvent, salt, and residual water were removed 20 fold faster than utilizing conventional methods. The porosities are uniform throughout the femoral shaped scaffold generated with PLA or PLA-HA. Both scaffolds demonstrated good biocompatibility with the pre-osteoblasts (MC3T3-E1) fully attaching to the scaffold within 8 h. The cells demonstrated high viability and proliferation throughout the entire time course. The HA-incorporated scaffolds demonstrated significantly higher compressive strength, modulus and osteoinductivity as evidenced by higher levels of alkaline-phosphatase activity and calcium deposition. When 3D printing a 3D model at 95% porosity or above, our technology preserves integrity and surface detail when compared with FDM-generated scaffolds. Our technology can also generate scaffolds with a 31 fold larger S/V than FDM. We have developed a technology that is a versatile tool in creating personalized, patient-specific bone graft scaffolds efficiently with high porosity, good scaffold integrity, high anatomical-shaped surface detail and large S/V.

## 1. Introduction

With an annual cost of more than $2.5 billion, diseases and trauma resulting in bone defects have affected 500,000 lives/year in the US and can be classified as a serious health problem [1]. Autografts are often utilized to heal these defects but result in donor site morbidity and are limited by the availability of the donor bone [2]. Allografts, such as demineralized bone matrix and cancellous chips, face potential immune rejection, the risk of communicable diseases and have lower incorporation rates [3]. Both autograft and allograft have limited capability to recreate the anatomical features of the bone [4]. Tissue engineering holds great potential to overcome these major drawbacks [5,6,7]. Encouraging progress in bone tissue engineering has been made in recent years. Different types of scaffold substrates have previously been used to generate engineered bone including decellularized bone, β-tricalcium phosphate (TCP), hydroxyapatite (HA), various hydrogels, and various polymers [8,9,10,11]. As a result of the many challenges in generating complex shapes with these materials, they have not been utilized for anatomically-shaped scaffolds. Regenerating the anatomical features of the native bone may benefit patients in regaining lost function more quickly. However, there have only been a few techniques reported for generating anatomically-shaped scaffolds. One technique is the computer-numerical-control (CNC) machining of an existing decellularized bone graft [12]. This method is limited by the bone graft availability and can only generate a small-sized scaffold because of material sacrifice during the machining process. 3D printing is another emerging technique to generate anatomically-shaped scaffolds [13,14,15,16,17,18]. Fused deposition modeling (FDM) is the most widely used 3D printing technique in tissue engineering [19,20,21,22]. Even though there are many commercially available FDM 3D printers on the market, these printers only print solid polymers such as polylactic acid polymer (PLA) and polycaprolactone (PCL). Scaffolds with high porosity, and large surface area-to-volume ratio are always desirable for tissue engineering [23]. One major limitation of FDM is that only low porosity [7] scaffolds can be fabricated while maintaining the integrity and a reasonable S/V. When HA particles are incorporated in the FDM printing process, the porosity is even lower because wider filament extrusion must be used to prevent clogging during polymer extrusion. Previous reports document only 30% porosity when PLA-HA scaffolds are constructed in this manner [24]. Porosity, integrity and S/V are critical features for bone scaffold construction because of their direct relationships with scaffold degradation, cell viability, cell proliferation, bone remodeling, and mechanical support. Solvent casting and particulate leaching (SCPL), which can generate a scaffold with over 90% porosity and large S/V, has previously been widely used in bone tissue engineering [25,26,27]. The process includes casting the dissolved polymer and soluble particles, such as sugar and salt, in a mold, evaporating the solvent and dissolving the particles in water. Various polymers can be used with this technique and the pore size can be easily controlled by altering the size of the particles [28,29]. To the best of our knowledge, there is no prior report on anatomically-shaped scaffold generation using SCPL. This is likely due to the difficulty in generating an anatomically-shaped mold. Teflon and ceramic have previously been used for these molds. However, Teflon cannot be used to generate anatomical shapes and ceramic requires a wax inverse-mold which increases the processing time and risks reducing accuracy. Additionally, they share a common limitation that the mold is difficult to remove after the scaffold is fabricated. Ideal materials for creating anatomically-shaped molds require strong solvent resistance, ease of fabrication and ease of removal [30,31].

Polyvinyl Alcohol (PVA), a thermal polymer, has been used in industry previously as a 3D printing material. It is chemically resistant and our preliminary tests show that it is very stable in organic solvents. An additional benefit is its water solubility. This allows it to be removed easily after polymer hardening. To the best of our knowledge, PVA has not been utilized as a molding material for anatomically-shaped scaffold fabrication even though it has been previously used for simple cubic shaped mold fabrication [32].

Removal of solvent, porogen particle, and residual water is time consuming in the SCPL process using conventional methodologies. Solvent and residual water removal rely on evaporation and the porogen particle removal relies on porogen static-dissolving in water. All of the processes are in static mode, therefore each takes a few days to accomplish even for small-sized scaffolds.

Here we report on our success fabricating anatomically-shaped bone scaffolds using a novel indirect 3D printing and perfusion (3D P&P) technique. This innovative approach utilizes the 3D printing of an anatomically-shaped mold using PVA, casting PLA or PLA-HA mixed salt particles in the organic solvent solution, and perfusion-based methods to rapidly remove solvent, porogen particles, and residual water. The generated scaffolds have identical shape characteristics of the imaged bone. The objective of the current study is to establish a novel 3D printing platform to generate anatomically-shaped scaffolds with high porosity instead of developing new materials. Here we specifically utilized PLA and hydroxyapatite as the materials because they have been widely used and well characterized previously [33,34], which makes them an appropriate tool to evaluate a new methodology. In addition, we also compared our 3D P&P technology with FDM in terms of scaffold porosity, external surface detail, integrity, and S/V.

## 2. Results

### 2.1. Anatomically-Shaped Digital Model Generation and Casting Mold Fabrication

**Figure 1:**
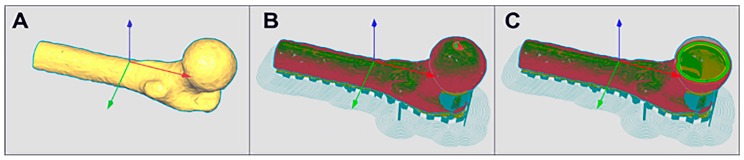
(**A**) The 3D femur model reconstructed from the CT images of a deidentified real patient. (**B**) The hollow mold model which was used to 3D print the Polyvinyl Alcohol (PVA) mold. (**C**) demonstrates the hollow interior space within the reconstructed model shown (**A**). The arrows indicate the X, Y and Z axes.

**Figure 2:**
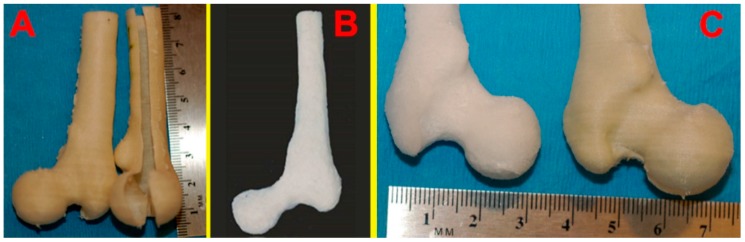
Two identical molds were successfully 3D printed as demonstrated in (**A**), one of which was sliced open to show the interior. The anatomically-shaped scaffold was also successfully generated as shown in (**B**) and it has the same detailed external anatomical characteristics as the mold (**C**).

An anatomically-shaped digital model of the proximal portion of the femur was successfully generated as shown in Figure 1. Figure 1A shows the 3D femur model reconstructed from the CT images of a deidentified real patient. Figure 1B shows the hollow mold model that was used to 3D print the PVA mold. Figure 1C demonstrates the hollow interior space within the reconstructed model shown in Figure 1A. There is no interior filling in the hollow mold, therefore supporting features were added in the digital mold model in order to stabilize the mold during the 3D printing process as shown in Figure 1A,B. Figure 2A demonstrates two identical successfully printed 3D molds, one of which was sliced open to show the interior. The anatomically-shaped scaffold was also successfully generated (Figure 2B) with the same detailed anatomical characteristics as the mold (Figure 2C). The surfaces of the scaffold were generated with the same level of smoothness as the original bone, by comparing the corresponding surfaces of the 3D model, which was reconstructed from the CT images, and the fabricated scaffold. The assumption that the information from the CT images represent those of the original bone surfaces was made.

As shown in Table 1, by perfusing with ethanol, water, and dry air sequentially, the solvent, salt, and residual water were quickly removed. Our perfusion-based approach has a processing time that is more than 20 fold faster than conventional methods.

### 2.2. Porosity and Micro-Architecture of the Scaffold

**Figure 3:**
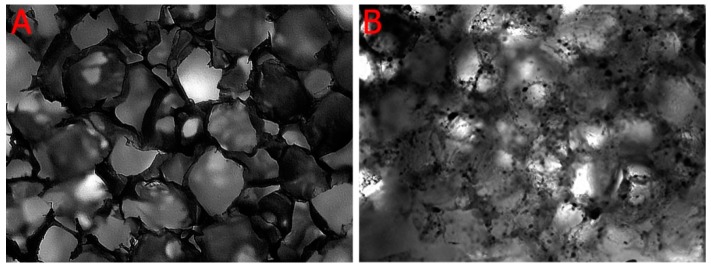
Phase contrast images show that both polylactic acid polymer (PLA) (**A**) and PLA-hydroxyapatite (HA) (**B**) scaffolds are highly porous and interconnected. Images were captured with a 4× objective.

As shown in Figure 3, the microscopic images demonstrate that both PLA (Figure 3A) and PLA-HA (Figure 3B) scaffolds are highly porous and interconnected. The results of the absolute alcohol displacement assay demonstrated porosities of the PLA and PLA-HA disc scaffolds to be 97.14% and 92.66%, respectively. The porosities of PLA and PLA-HA sections from the femoral head, femoral neck, proximal and distal portions of the femoral shaft are shown in Table 2. There were no significant differences among the different locations of the anatomically-shaped scaffold and no porosity differences between simple shaped disc scaffolds and complex anatomically-shaped scaffolds when the same size of leaching particles were used. This demonstrated that our technique was able to generate complex anatomically-shaped scaffolds with evenly distributed porosities.

### 2.3. Cell Viability and Proliferation

**Figure 4:**
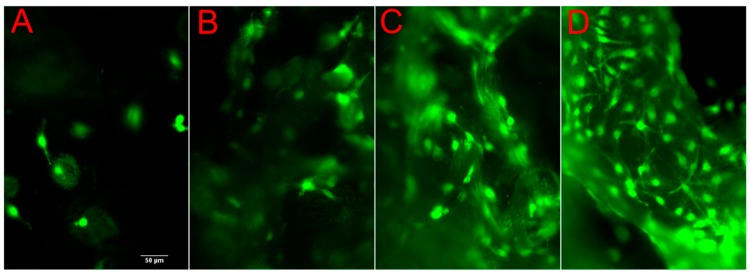
The cells demonstrated high viability throughout the period of observation as demonstrated by Calcein AM staining. Representative images of cells grown on PLA-HA scaffold are shown. (**A**) day 1, (**B**) day 3, (**C**) day 5, (**D**) day 7). Bar equals to 50 µm and applied to all the images.

**Figure 5:**
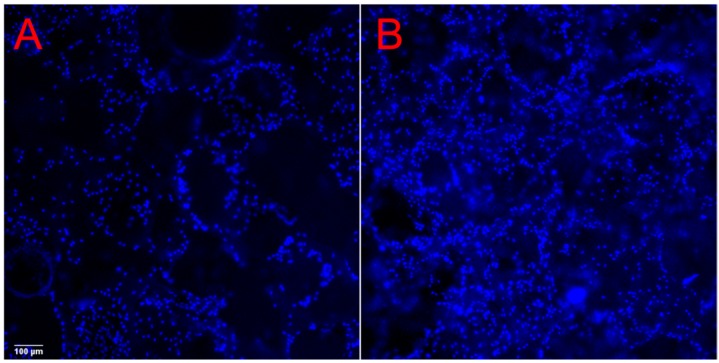
A higher density of cells was observed on the PLA-HA scaffold (**B**) than on the PLA scaffold (**A**) as shown by epi fluorescent imaging of 4′,6-diamidino-2-phenylindole (DAPI) staining (images were obtained after five days of culture using a lower magnification objective for a larger field of view).

**Figure 6:**
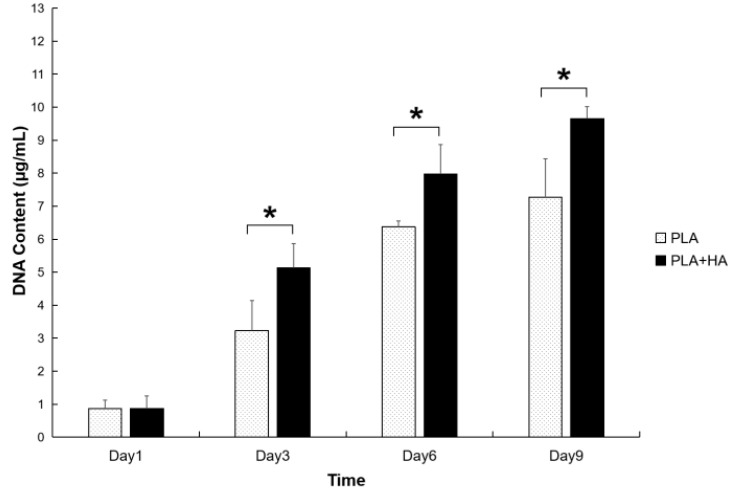
Proliferation chart shows that from the quantitative results of DNA content analysis, both types of scaffolds support cell growth with the PLA-HA group showing a significantly higher proliferation rate than the PLA group starting from day 3 to day 9. * indicates *p* < 0.05.

The cells remained highly viable throughout the period of observation as demonstrated by Calcein AM staining (Figure 4A–D, day 1–7). The cell viability of cells grown on both PLA and PLA-HA scaffolds show over 90% cell viability. A higher density of cells was observed on the PLA-HA scaffold than on the PLA scaffolds as shown by epi fluorescent imaging of 4′,6-diamidino-2-phenylindole (DAPI) staining (Figure 5, day 5). This implies that there are pro-proliferative effects of the incorporated HA on pre-osteoblasts. From the quantitative results of DNA content analysis (Figure 6), the PLA-HA group shows a significantly higher proliferation rate than the PLA group starting from day 3 to day 9, which further confirms the pro-proliferative effects of the incorporated HA.

### 2.4. ALP Activities

**Figure 7:**
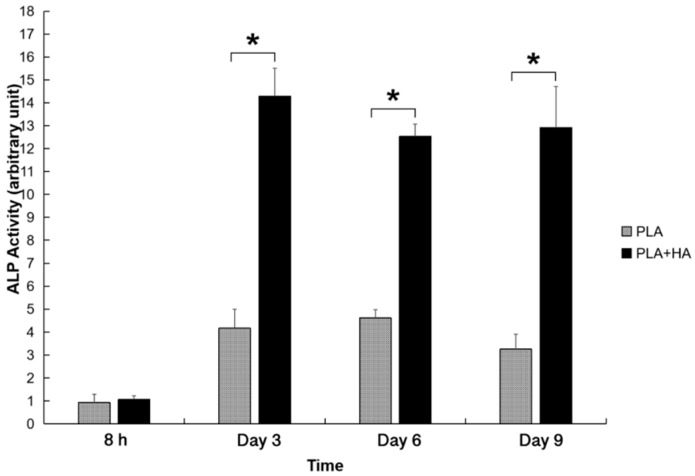
HA-incorporated scaffold demonstrated significantly higher osteoinductivity than PLA only scaffold as demonstrated by the Alkaline-Phosphatase (ALP) activity. * indicates *p* < 0.01.

Both PLA and PLA-HA scaffolds provided a 3D environment that supported MC3T3-E1 to undergo osteogenic differentiation. As shown in Figure 7, both PLA and PLA-HA scaffolds show increased Alkaline-Phosphatase (ALP) activities over the time course evaluated. The ALP activities in the PLA-HA and PLA groups reached a peak value at day 3 and day 6, respectively. Cells growing within the PLA-HA scaffold demonstrated significantly higher ALP activates than the PLA scaffold (*p* < 0.01). This was due to the incorporation of HA which demonstrated higher levels of osteoinductivity. At day 3, the ALP activities in the PLA-HA was more than 3 times higher than those in the PLA group.

### 2.5. Calcium Deposition

**Figure 8:**
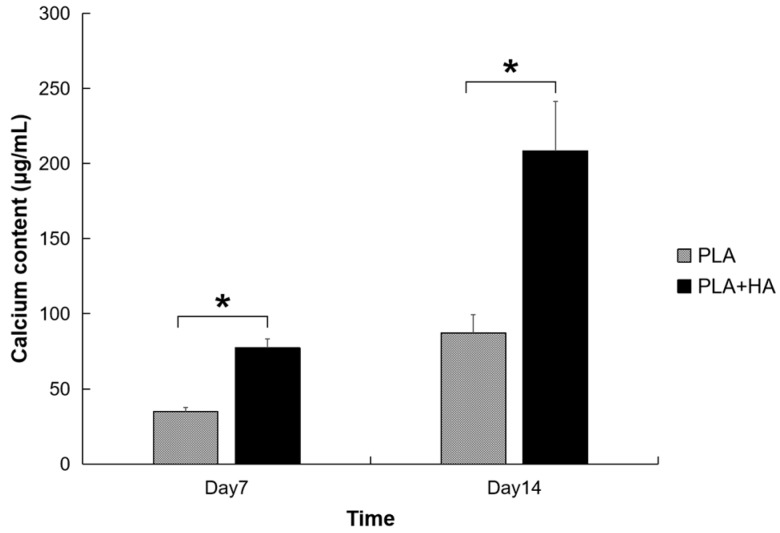
HA-incorporated scaffolds show significantly higher calcium deposition than the PLA-only scaffold. * indicates *p* < 0.01.

Similar to the osteodifferentiation activity observed when using ALP as a biomarker, the pre-osteoblasts show increased activity of calcium deposition (Figure 8) when cultured within both the PLA and PLA-HA scaffolds. Both ALP and calcium deposition are biomarkers of cell osteodifferentiation with calcium deposition being more specific than ALP activity. Similar to the osteoinductivity of the HA observed in the assay of ALP activity, HA-incorporated scaffolds show significantly higher calcium deposition than the PLA-only scaffold (*p* < 0.01).

### 2.6. Mechanical Analysis

**Figure 9:**
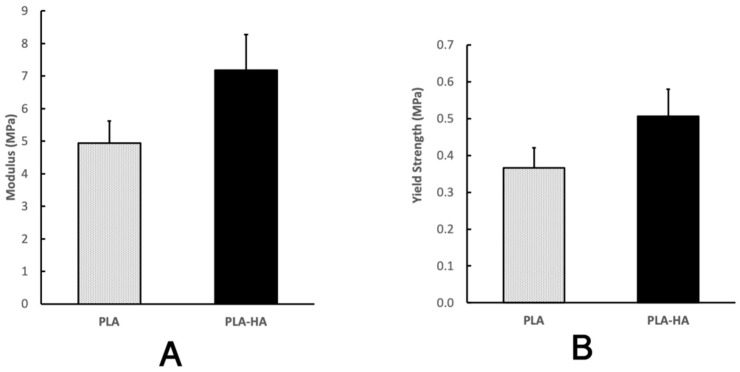
HA incorporated porous scaffold shows significantly higher compressive modulus (**A**) and strength (**B**) than the PLA only scaffold (*p* < 0.01).

As shown in Figure 9, the yield strengths of the PLA and PLA-HA porous scaffolds were 0.366 ± 0.055 MPa and 0.506 ± 0.073 MPa, respectively. The compressive moduli of PLA and PLA-HA porous scaffolds were 4.935 ± 0.687 MPa and 7.182 ± 1.096 MPa respectively. The 20% of HA incorporation significantly increased the yield strength and the compressive modulus by 38% and 46%, respectively (*p* < 0.01).

### 2.7. Comparison Assay with FDM Technology

**Figure 10:**
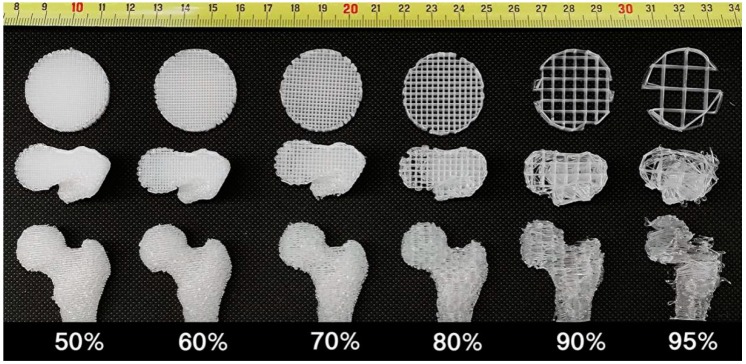
From left to right, images of scaffolds with 50%, 60%, 70%, 80%, 90%, and 95% porosities generated by fused deposition modeling (FDM) technology. Top and bottom rows show the scaffolds 3D printed using the disk model and anatomically-shaped femur model, respectively. The middle row shows the internal structures of the scaffolds of the bottom row.

As demonstrated in the disk models in Figure 10, in order to achieve higher porosity, strut distance must be increased, resulting in less filament deposition and shorter total fiber length. In turn, this results in smaller S/V. In the anatomical-shaped model as shown in Figure 10 and Table 3, FDM-scaffolds show lower print resolution with less smooth surfaces at the 70% porosity and rough surfaces at 80% porosity. This is because increased strut distance results in less surface detail. At 90% porosity, scaffold integrity loss appeared and the anatomical-shape outlines in the printed model shows deformation compared with the original 3D digital model. At 95% porosity, the integrity is lost almost completely and the anatomical-shape of the original 3D model is poorly reproduced. Compare this to the shape characteristics and 97% average porosity of the scaffolds generated using 3D P&P technology with only PLA material. As noted in Table 3, when the porosity in the FDM-scaffold increases, the surface smoothness and S/V decrease. When the porosity reaches 95%, the surface detail is lost dramatically and S/V drops to 0.5. This is 31 times less than that of the scaffolds generated using 3D P&P at 97% porosity. Printing at high porosity using FDM technology results in poor surface detail, poor surface integrity and low S/V, while the 3D P&P technology can generate scaffolds with high porosity, high anatomical-shape surface detail, high integrity and large S/V.

## 3. Discussion

Even for the latest high-end 3D FDM printers, the mechanical resolution of the X, Y, and Z axes, which can be as low as several microns, must not be confused with the smallest element that a printer can generate. This parameter is dictated by the diameter of the thread extruded from the printer head and ranges from a few hundred microns to several hundred microns, and even larger when HA is incorporated into the polymer. These limitations do not allow FDM 3D printing to generate high porosity scaffolds. Mixing micro-particles, such as HA, with the polymer is also a time consuming and challenging pre-printing process. Most commercially available polymers do not come in powder form, so polymer grinding or heat mixing is required. As demonstrated in the current project, and in previous studies [20,35,36], porosity contradicts surface detail, integrity, and S/V. This fact explains why many scaffolds fabricated by FDM previously utilized low porosity in order to balance the other parameters. These limitations of FDM scaffold printing, especially with regard to particle incorporation, beckon the need for new methodologies.

Particle leaching is a low cost, simple method to generate scaffolds with high porosity. However, a significant drawback to this technique is the challenge of generating anatomically-shaped scaffolds. This is primarily due to the difficulty generating specific complex shaped molds with the molding materials used previously [27]. Our novel 3D P&P approach overcomes this limitation by 3D printing hollow molds using PVA material which is solvent-resistant but can be easily removed after scaffold generation.

Image processing is a critical step in generating anatomically-shaped scaffolds. In order for our 3D P&P technology to have a broader contribution to the scientific community, we primarily used open source software to perform the image processing. Due to the complex shape of bones, internal supporting features need to be added so that the mold does not collapse during printing. In order to generate a smooth internal surface, a 100 µm layer height is preferred and the printing speed should not exceed 100 mm/s. Based on our experience, a minimum 2 mm wall thickness should be used for the mold to prevent any leakage.

Removing solvent, porogen, and residual water from the scaffolds are also important steps in the SCPL method. Conventionally, these compounds were removed passively with the solvent and water removal occurring through evaporation and porogen removal carried out through a dissolving process. The entire process usually takes several days. For relatively large scaffolds, removing the porogen in the center can be even slower. Ultrasonography has been utilized to assist the dissolving process. However, our experience (data not shown) has been that ultrasound only speeds up the dissolving process to a limited degree [37]. As the ultrasonic waves heat the water, there is an adverse impact on the microscopic architecture of the scaffold. Instead of using static methods, we created a novel dynamic approach to allow rapid removal of the compounds. The key to this process is that dichloromethane is miscible in ethanol. The flowing ethanol utilized in our technique removes the dichloromethane quickly. Evaporation-based methods require a minimum of 2 days to fully solidify the polymer even for a small scaffold. During this period, the salt and HA tend to gravitate to the bottom of the mold resulting in an uneven distribution of both HA and pores. Another benefit of rapid solvent removal is solidifying the polymer quickly so that the salts and HA particles are evenly distributed. For this step, a low-flow-rate perfusion should be applied until the PLA polymer solidifies. This allows the distribution of the porogen and the HA to not be disrupted. After the initial ethanol perfusion, a high flow rate can be applied to quickly and thoroughly remove the residual solvent. Some pressure-control should be employed to remove the salt by perfusion. Perfusion can quickly remove the salt by accelerating its dissolution. Additionally, this process does not require the salt particles to be fully dissolved because it flushes out the salt particles when they get small enough to pass through the pores. Conventional techniques require the porogen-free scaffold to be oven-dried for up to 48 h to remove the residual water within it [2]. By using perfused dry air in our approach, we were able to remove the residual water within 10 min, even for larger-sized scaffolds. The salt particles are packed inside the mold, therefore the solution of dissolved polymer filled in the micro-spaces between particles. As the alcohol flows through the micro-spaces to remove the solvent, the polymer solidified locally within the micro-space. Therefore, there is no global volume shrinkage.

Our 3D P&P approach allows production of a highly porous scaffold with the porosity evenly distributed throughout the structure, even within a complex anatomically-shaped scaffold. Additionally, microscopic evaluation from multiple locations within samples also reveals an even distribution of HA particles.

Biocompatibility is an important feature for any scaffold. Osteoinductivity, which means that undifferentiated cells are stimulated to develop into a bone-forming cell lineage [38], is an equally important feature for scaffolds used for bone regeneration [39]. The scaffolds generated using the 3D P&P technique are highly biocompatible as evidenced by the observation that cells grown on these scaffolds demonstrated high viability and continued to proliferate throughout the entire course of the experiment. At the conclusion of the experiment, the entire scaffold was populated with MC3T3-E1 cells with elongated cell bodies. There were no significant differences in the cell viability among the cells grown on the PLA, PLA-HA, and tissue culture dishes. This indicates that the technique successfully and thoroughly removes the toxic solvent. Our results show that HA-incorporated scaffolds promoted cell proliferation at a higher rate than PLA scaffolds. Similar results have been reported previously [3]. This is likely due to the proliferation of osteoblasts being linked to the intrinsic rigidity and the micro-architecture of the substrate [40].

Hydroxyapatite has also previously been reported to support osteodifferentiation when incorporated into various polymers [4,5]. PLA-HA scaffolds fabricated using 3D P&P technology demonstrated significantly greater osteoinductivity than the PLA-only scaffolds as indicated by higher levels of ALP activity and calcium deposition. The concentration of HA (20%) was chosen here because it was the concentration utilized in previous studies [26,41] with improved biological and biomechanical functions. A future study might include varying concentrations of HA for further optimization.

While HA incorporation significantly improved the mechanical properties of the generated porous scaffold, it remains lower than those of a native bone. This has been reported previously when using the conventional SCPL method [26,42]. The ultimate goal of the current project is to generate an anatomically-shaped precursor bone construct with reasonable weight-bearing capability in vitro which has the capacity to mature into the full weight-bearing bone in vivo according to Wolff’s law We anticipate that when it comes to a clinical use, the scaffold will need to be implanted in a load-sharing rather than in a load bearing manner.

One challenge of culturing cells in a relatively large scaffold is the difficulty of delivering nutrition to the cells. In this study, we utilized perfusion to remove solvent, porogen, and residual water from our scaffolds. Using these same principles, we are currently studying perfusing culture media through the anatomically-shaped scaffold while covered with a 3D printed water-tight shell of the same shape.

There are limitations inherent in the current project. As this is a proof of concept study, we used a scaled down hollow mold and fabricated the scaffolds with uniform internal structure and porosity. As with previous studies that focused on producing “anatomically-shaped scaffolds”, we focused on recapitulating the outer anatomic shape of the bone rather than the internal structure [12,17]. Future studies can focus on other anatomical features in addition to shape, such as internal structure, which can be created using 3D printed multiple-compartmented molds so that different porogens and polymers can be casted and leached. The physical and biomechanical alterations as a function of degradation in vitro and in vivo should be included in future studies. In addition, as a consequence of PLA and PLA-HA scaffold degradation, acid and iron release occurs. Other polymers without acid release and the release profiles of calcium and phosphorus ions from the scaffold should be investigated. Translation of this 3D P&P technology into the clinical realm, like any other translational tissue engineering technologies, must consider manufacturing, regulatory compliance, hospital procurement, and reimbursement [43,44].

In this study, we developed a novel indirect 3D printing and perfusion techniques to successfully fabricate anatomically-shaped custom scaffolds to precisely match a patient’s femural bone. We were able to accelerate the fabrication process from 12 to 192 times when compared to conventional methods. The fabricated scaffolds are highly porous and demonstrate uniform porosity throughout the scaffold. These scaffolds are also biocompatible and osteoinductive. Their mechanical compressive strength was significantly increased with HA incorporation. When compare with FDM technology printed at high porosity levels, the 3D P&P scaffolds maintained high structural integrity and anatomical-shape detail as well as large S/V.

## 4. Materials and Methods

### 4.1. Generating Anatomically-Shaped 3D Model

De-identified computed tomography (CT) images of a healthy human femur were obtained. The femur model was chosen because it represented a relatively complex anatomical-shape of bone. The digital images were imported into an open source 3D slicing software (Slicer, Boston, MA, USA) to generate a solid stereolithography (STL) 3D file. The hard tissue threshold was employed so that only the target bone was selected to generate the 3D model. A mirror process was then performed so that final 3D model could theoretically be used to repair a defect in the patient’s contralateral site. The generated STL was further processed in the open source Blender software (Blender, Amsterdam, Netherlands) to generate a hollow 3D model. Perfusing inlets and outlets were designed as necessary at the appropriate positions. The generated 3D model was adjusted to the height of 70 mm in proportion to the original dimensions. Open source CURA software was used to generate machine codes (Gcodes).

### 4.2. Mold Generation by 3D Printing

A custom developed Fused Deposition Modeling (FDM) 3D printer [45,46] was used to print the mold. The PVA (Flashforge USA, City of Industry, CA, USA) material was melted at 180 °C and extruded through a 0.3 mm nozzle with a layer height of 0.1 mm. A 2 mm thick PVA wall was printed. A two-parts mold that tightly conformed to the PVA mold was printed using the acrylonitrile butadiene styrene (ABS, Flashforge, USA) material. The ABS mold was used for porogen particles and residual water removal.

### 4.3. Scaffold Manufacture

Poylactic acid polymer (PLA, NatureWorks, Minnetonka, MN, USA) material at a density of 1.25 g/cm^3^ was dissolved in dichloromethane (Sigma-Aldrich, St. Louis, MO, USA) to a concentration of 10% (*w*/*v*) and shaken gently overnight. Sodium chloride (NaCl, Fisher Scientific, Pittsburgh, PA, USA) was sieved to obtain a particle size 300–500 µm. The fully dissolved PLA solution was then mixed with the sieved NaCl particles and casted into the 3D printed PVA anatomically-shaped 70 mm long femoral mold (Figure 10). For the PLA-hydroxyapatite composite (PLA-HA) scaffold, dissolved PLA was mixed with 20% (*w*/*v*) hydroxyapatite powder (less than 10 μm in particle size, from Hitemco Medical, Old Bethpage, NY, USA). The mixture was further combined with NaCl as detailed above and poured into the mold.

**Figure 11:**
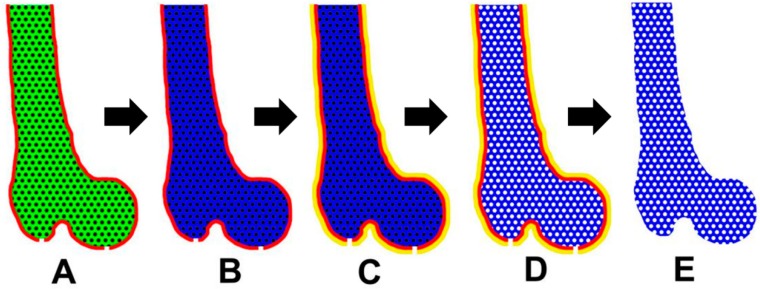
Schematics of the workflow. (**A**) The PLA polymer was dissolved in dichloromethane (green indicates the PLA solution) and mixed with NaCl (black) and loaded into the PVA mold (red). (**B**) The dichloromethane was removed by perfusing with 100% ethanol (blue indicates the solid PLA). (**C**) A 3D printed mold using acrylonitrile butadiene styrene (ABS, yellow) was added to cover the original PVA mold. (**D**) NaCl porogens were removed by perfusing with water. (**E**) An anatomically-shaped scaffold was generated after removing the ABS and PVA molds (white indicates the pores).

Next, the mold was connected to a perfusion system through the designed ports. A 50:1 volume of ethanol:solvent was added from the top opening (Figure 11A) and a vacuum was applied from the bottom so that the ethanol flowed through the polymer to remove the solvent. The level of vacuum is preoptimized according to the size and shape of the scaffold. It was controlled to increase gradually by monitoring the level of fluid passing through the scaffold in order to minimize the deformation of the scaffold by the vacuum pressure. The molded polymer solidified after removing the solvent. A 750:1 volume of distilled water: solvent was perfused through the material to remove the residual ethanol and NaCl particles. Most of the PVA layer was dissolved during the perfusion after about 40% of the total amount of water was used. Next, the construct was soaked in a stirred distilled water bath to quickly remove any remaining PVA mold. The wet scaffold was placed into another conforming two part anatomically-shaped ABS mold that was independently printed previously and the remaining 60% of water was further perfused. Finally, filtered dry air was perfused through the construct in a dust-free hood to completely remove any residual water, which was confirmed when there was no further weight decrease.

A 70 mm long × 10 mm diameter 3D printed PVA cylindrical mold was used to generate a cylinder scaffold using the same protocol as above. The generated scaffold was sliced into disks with 5 mm height using a custom-fabricated two blade cutter for biocompatibility analysis.

In order to compare the processing time of removing solvent, salt, and residual water between our perfusion-based approach and the conventional method, 3D printed cylinder molds were used to cast a polymer of approximately 15 mm diameter × 7 mm height. After polymer casting, the removal of solvent, salt, and residual water was accomplished using our perfusion approach and the conventional method [42] independently, and the processing time for each step was recorded.

### 4.4. Micro-Architecture and Porosity Analysis

To examine the microarchitecture, disc shaped (10 mm diameter × 5 mm height) scaffolds (PLA and PLA-HA) were created with disk molds using the same methods as documented above. The disk scaffold was examined using a Nikon inverted microscope (Eclipse TE2000, Nikon, NY, U.S.A.) and images were obtained using a Retiga camera (Teledyne QImaging, Surrey, Canada). In order to analyze whether there were differences in porosity in the 70 mm femur-shaped scaffold, 5 mm slices were obtained and imaged from four locations: the femoral head, femoral neck, proximal femoral shaft, and distal femoral shaft. The porosity of the slices was measured using an absolute alcohol displacement assay according to a protocol published previously [41,47].

### 4.5. Cell Culture and Seeding into the Scaffolds

Preosteoblast MC3T3-E1 cells (ATCC) were grown in Dulbecco’s Modified Eagle’s Medium (DMEM, Life Technology, Carlsbad, CA, USA) with 10% FBS at 37 °C with 5% CO_2_. Medium was changed 2–3 times per week. Disk scaffolds were prepared as above and coated with 100 µg/mL bovine collagen solution (PureCol^®^, Advanced BioMatrix, Inc. San Diego, USA). One piece of scaffold was placed in each well of a 24-well plate. Once they reached approximately 80% confluence, cells were detached with 0.05% trypsin-EDTA (Life Technology) and adjusted to 1 × 10^5^/mL. Five-hundred microliters of cell suspension was seeded onto each scaffold and cultured in the incubator at 37 °C with 5% CO_2_. After 2 h, 500 µL complete culture medium was added into each well and cultured under the same conditions.

### 4.6. Cell Viability Analysis

Cell viability was analyzed using a live/dead cell staining kit according to instructions from the manufacturer (Thermo Fisher Scientific) [48]. In brief, 1 mL DMSO and 9 mL DMEM media without serum were mixed with 50 µg Calcein-AM and part B of the kit to generate the staining solution. The disk-shaped scaffolds seeded with MC3T3-E1 were washed 3 times with PBS and stained using the above solution for 30 min in the dark. Cell viability was then observed under a Nikon epifluorescence microscope (Eclipse TE2000-U) and images were obtained. The cytoplasms of the live cells demonstrate green fluorescence and the nuclei of dead cells show red fluorescence. The images were further processed and analyzed using ImageJ software (Rasband, W.S., ImageJ, U.S. National Institutes of Health, Bethesda, MD, USA, https://imagej.nih.gov/ij/, 1997–2018.)

### 4.7. Cell Proliferation Analysis

A cell proliferation analysis was carried out using a PicoGreen dsDNA quantification kit (Invitrogen) according to instructions from the manufacturer. In brief, the scaffolds were rinsed with PBS and the cells within the scaffolds were lysed with a lysis solution (0.1% (*v*/*v*) Triton X-100, 10 mm Tris, 1 mm EDTA) followed by three freeze-thaw cycles. 100 µL of DNA solution was mixed with an equal volume of PicoGreen working solution and added into each well of a 96-well plate. The fluorescence intensity of the mixed solution was analyzed using a fluorescence plate reader (Wallac Victor3, Perkin Elmer, MA, USA).

### 4.8. Alkaline-Phosphatase (ALP) Activity Assay

The MC3T3-E1 cells were cultured on scaffolds in the medium as above with supplementary 50 µg/mL ascorbic acid, 10 nM dexamethasone, and 10 nM β-glycerolphosphate added. The ALP activities were analyzed at days 3, 6, and 9 (3 scaffolds per group per time point). For each analysis, the scaffolds were rinsed with PBS and lysed. The reaction solution consisting of 2 mg/mL p-nitrophenyl phosphate (Sigma) and 0.1 M amino propanol (10 µL/well) in 2 mM MgCl_2_ (100 µL/well) at a pH 10.5, was added to each scaffold and incubated at 37 °C for 15 min. The reaction was stopped with 50 mM NaOH and the result was obtained from the reading of the absorbance at 410 nm using a plate reader. The DNA content of the cells was quantified using a PicoGreen dsDNA quantification kit as above. The results of ALP activity were recorded as ng ALP per μg total DNA content. The ALP activity of the cells seeded onto the PLA scaffold after only 8 h, which is the time point when maximal cell attachment was reached, was recorded as the baseline. All of the data was further normalized to this baseline.

### 4.9. Calcium Content Measurement

Scaffolds were seeded and cultured as in the ALP activity assay described above. After 7 and 14 days, the scaffolds were washed twice with PBS and incubated in 0.5 N acetic acid solution in an orbital shaker overnight. The calcium content was quantified using the OCPC (orthocresolphthalein complex one) method as reported previously [49]. Briefly, 20 μL of the samples were incubated with 250 μL of the working solution consisting of 0.05 mg/mL OCPC solution and ethanolamine/boric acid/8-hydroxyquinoline buffer (Sigma) in a 96-well plate for 10 min at room temperature and the plate was read using a microplate reader (PerkinElmer, MA) at 570 nm. CaCl_2_ solutions with known concentrations were used as the standard for the assay. In order to calculate the calcium deposition on the PLA-HA, the scaffolds without cells were incubated in the medium and the amount of calcium was measured as the baseline. The calcium deposited by the cells was the difference between the total amount of calcium and the baseline.

### 4.10. Mechanical Property Analysis

The compressive mechanical properties of the PLA (*n* = 5) and PLA/HA (*n* = 5) porous cylinder scaffolds were analyzed at the room temperature using a universal testing machine, which is comprised of a ball-screw guide rail driven by a servo motor (Panasonic, Osaka, Japan) and a displacement encoder with the resolution of 0.5 µm (Panasonic, Japan) and load cells (HBM, Suzhou, China) and custom developed compress platens. The scaffold was compressed between two stainless steel platens at the rate of 1 mm/min and data was recorded using 200 Hz sampling frequency. According to ASTM F451–16, the stress-strain curves were generated, and the slopes of the initial linear portions were used to calculate the values of compressive modulus and the values of compressive strength were calculated from the peak of the curve.

### 4.11. Comparison with FDM Technology

Fibrous scaffolds were 3D printed using the same 3D model and 3D printer in FDM mode as documented above. A disk model of 30 mm in diameter and 5 mm in height was also printed in FDM mode for analysis. The 0.4 mm fiber diameter was used as it was widely reported previously as an optimized parameter. The porosities of 50%, 60%, 70%, 80%, 90%, and 95% were used to control the strut distance-this methodology was approved previously by both mathematical modeling and micro-CT analysis [36]. It is notable here that any scaffold designed with porosity higher than 95% failed to print. The porosity, anatomical-shape surface detail, scaffold integrity and S/V were compared between our 3D P&P technology and the FDM technology. Only PLA material was used in the comparison because of technical difficulties of generating anatomically-shaped scaffolds at higher porosity with the HA incorporation [36]. The porosity of the FDM-scaffold and the S/V of scaffolds fabricated using both technologies were calculated using the methods reported previously [35,50]. Surface details were evaluated as documented above and categorized by two senior scientists.

### 4.12. Statistical Analysis

Data are presented as mean ± standard deviation (SD). Independent t-test was used to analyze differences between two groups. Analysis of variance (ANOVA) was used to analyze overall differences among groups. Statistical significance was set at *p* < 0.05.

## Figures and Tables

**Table 1:** Processing time comparison between perfusion and conventional methods.

Processing Time	Perfusion (perf.)	Conventional Methods (conv.)	Time Ratio (Tperf./Tconv.)
Solvent Removal	15 min	48 h	1/192
Salt particle Removal	4 h	48 h	1/12
Residual water removal	10 min	10 h	1/60

By perfusing with ethanol, water, and dry air sequentially, the solvent, salt, and residual water were quickly removed. Our perfusion-based approach has a processing time that is more than 20 fold faster than conventional methods.

**Table 2:** Porosity analysis among different locations within the anatomically-shaped scaffolds.

Porosity Analysis	Femoral Head	Femoral Neck	Proximal Femoral Shaft	Distal Femoral Shaft
PLA	97.31 ± 0.85	96.80 ± 1.79	96.36 ± 0.86	97.38 ± 0.95
PLA-HA	92.02 ± 2.24	92.62 ± 0.43	90.78 ± 1.71	92.75 ± 2.08

There were no significant differences among the different locations of the anatomically-shaped scaffolds when the same size leaching particles were used. PLA, polylactic acid polymer; PLA-HA, PLA-hydroxyapatite.

**Table 3:** Comparison between FDM Technology and 3D P&P Technology.

	FDM Technology						3D P&P
Porosity	50%	60%	70%	80%	90%	95%	97%
Integrity	H	H	H	M	L	EL	H
Surface Detail	H	H	M	L	EL	EL	H
S/V	5.0	4.0	3.0	2.0	1.0	0.5	15.5

When the porosity in the FDM-scaffold increases, the surface smoothness and S/V decrease. When printing at high porosity, FDM technology cannot keep surface details at a high level or good integrity or large S/V, while 3D printing and perfusion (3D P&P) technology can generate scaffolds with high porosity, high anatomical-shape surface detail, good integrity, and large S/V. H = high, M = medium, L = low, EL = extreme low.

## Abbreviations

PLA	polylactic acid polymer
HA	hydroxyapatite
TCP	β-tricalcium phosphate
CNC	computer-numerical-control
FDM	Fused deposition modeling
SCPL	Solvent casting and particulate leaching
PVA	Polyvinyl Alcohol
CT	computed tomography
STL	solid stereolithography
ABS	acrylonitrile butadiene styrene
PCL	polycaprolactone
S/V	surface area-to-volume ratio

## References

[1] Desai B.M. (2007). Osteobiologics. Am. J. Orthop. (Belle Mead NJ).

[2] Athanasiou V.T., Papachristou D.J., Panagopoulos A., Saridis A., Scopa C.D., Megas P. (2010). Histological comparison of autograft, allograft-DBM, xenograft, and synthetic grafts in a trabecular bone defect: An experimental study in rabbits. Med. Sci. Monit..

[3] Oryan A., Alidadi S., Moshiri A., Maffulli N. (2014). Bone regenerative medicine: Classic options, novel strategies, and future directions. J. Orthop. Surg. Res..

[4] Calcei J.G., Rodeo S.A. (2019). Orthobiologics for Bone Healing. Clin. Sports Med..

[5] Amini A.R., Laurencin C.T., Nukavarapu S.P. (2012). Bone tissue engineering: Recent advances and challenges. Crit. Rev. Biomed. Eng..

[6] Wubneh A., Tsekoura E.K., Ayranci C., Uludag H. (2018). Current state of fabrication technologies and materials for bone tissue engineering. Acta Biomater..

[7] Hutmacher D.W. (2000). Scaffolds in tissue engineering bone and cartilage. Biomaterials.

[8] Lane J.M., Sandhu H.S. (1987). Current approaches to experimental bone grafting. Orthop. Clin. N. Am..

[9] Shin H., Jo S., Mikos A.G. (2003). Biomimetic materials for tissue engineering. Biomaterials.

[10] Silva G.A., Coutinho O.P., Ducheyne P., Reis R.L. (2007). Materials in particulate form for tissue engineering. 2. Applications in bone. J. Tissue Eng. Regen. Med..

[11] Li J., Baker B.A., Mou X., Ren N., Qiu J., Boughton R.I., Liu H. (2014). Biopolymer/Calcium phosphate scaffolds for bone tissue engineering. Adv. Healthc. Mater..

[12] Grayson W.L., Frohlich M., Yeager K., Bhumiratana S., Chan M.E., Cannizzaro C., Wan L.Q., Liu X.S., Guo X.E., Vunjak-Novakovic G. (2010). Engineering anatomically shaped human bone grafts. Proc. Natl. Acad. Sci. USA.

[13] Giordano R.A., Wu B.M., Borland S.W., Cima L.G., Sachs E.M., Cima M.J. (1996). Mechanical properties of dense polylactic acid structures fabricated by three dimensional printing. J. Biomater. Sci. Polym. Ed..

[14] Cooke M.N., Fisher J.P., Dean D., Rimnac C., Mikos A.G. (2003). Use of stereolithography to manufacture critical-sized 3D biodegradable scaffolds for bone ingrowth. J. Biomed. Mater. Res. B Appl. Biomater..

[15] Seitz H., Rieder W., Irsen S., Leukers B., Tille C. (2005). Three-dimensional printing of porous ceramic scaffolds for bone tissue engineering. J. Biomed. Mater. Res. B Appl. Biomater..

[16] Ge Z., Tian X., Heng B.C., Fan V., Yeo J.F., Cao T. (2009). Histological evaluation of osteogenesis of 3D-printed poly-lactic-co-glycolic acid (PLGA) scaffolds in a rabbit model. Biomed. Mater..

[17] Temple J.P., Hutton D.L., Hung B.P., Huri P.Y., Cook C.A., Kondragunta R., Jia X., Grayson W.L. (2014). Engineering anatomically shaped vascularized bone grafts with hASCs and 3D-printed PCL scaffolds. J. Biomed. Mater. Res. A.

[18] Kang H.W., Lee S.J., Ko I.K., Kengla C., Yoo J.J., Atala A. (2016). A 3D bioprinting system to produce human-scale tissue constructs with structural integrity. Nat. Biotechnol..

[19] Guo R., Merkel A.R., Sterling J.A., Davidson J.M., Guelcher S.A. (2015). Substrate modulus of 3D-printed scaffolds regulates the regenerative response in subcutaneous implants through the macrophage phenotype and Wnt signaling. Biomaterials.

[20] Nyberg E., Rindone A., Dorafshar A., Grayson W.L. (2017). Comparison of 3D-Printed Poly-varepsilon-Caprolactone Scaffolds Functionalized with Tricalcium Phosphate, Hydroxyapatite, Bio-Oss, or Decellularized Bone Matrix. Tissue Eng. Part A.

[21] Sa M.W., Nguyen B.B., Moriarty R.A., Kamalitdinov T., Fisher J.P., Kim J.Y. (2018). Fabrication and evaluation of 3D printed BCP scaffolds reinforced with ZrO2 for bone tissue applications. Biotechnol. Bioeng..

[22] Cheng C.H., Chen Y.W., Lee A.K.-X., Yao C.H., Shie M.Y. (2019). Development of mussel-inspired 3D-printed poly (lactic acid) scaffold grafted with bone morphogenetic protein-2 for stimulating osteogenesis. J. Mater. Sci. Mater. Med..

[23] Lu L., Mikos A.G. (1996). The importance of new processing techniques in tissue engineering. Bulletin.

[24] Senatov F.S., Niaza K.V., Zadorozhnyy M.Y., Maksimkin A.V., Kaloshkin S.D., Estrin Y.Z. (2016). Mechanical properties and shape memory effect of 3D-printed PLA-based porous scaffolds. J. Mech. Behav. Biomed. Mater..

[25] Liao C.J., Chen C.F., Chen J.H., Chiang S.F., Lin Y.J., Chang K.Y. (2002). Fabrication of porous biodegradable polymer scaffolds using a solvent merging/particulate leaching method. J. Biomed. Mater. Res..

[26] Wei G., Ma P.X. (2009). Partially nanofibrous architecture of 3D tissue engineering scaffolds. Biomaterials.

[27] Suh S.W., Shin J.Y., Kim J., Kim J., Beak C.H., Kim D.I., Kim H., Jeon S.S., Choo I.W. (2002). Effect of different particles on cell proliferation in polymer scaffolds using a solvent-casting and particulate leaching technique. ASAIO J..

[28] Mikos A.G., Thorsen A.J., Czerwonka L.A., Bao Y., Langer R., Winslow D.N., Vacanti J.P. (1994). Preparation and characterization of poly (L-lactic acid) foams. Polymer.

[29] Mikos A.G., Sarakinos G., Leite S.M., Vacant J.P., Langer R. (1993). Laminated three-dimensional biodegradable foams for use in tissue engineering. Biomaterials.

[30] Okada K., Hasegawa F., Kameshima Y., Nakajima A. (2007). Bioactivity of CaSiO 3/poly-lactic acid (PLA) composites prepared by various surface loading methods of CaSiO 3 powder. J. Mater. Sci. Mater. Med..

[31] Chen J.-L., Chiang C.-H., Yeh M.-K. (2002). The mechanism of PLA microparticle formation by waterin-oil-in-water solvent evaporation method. J. Microencapsul..

[32] Mohanty S., Larsen L.B., Trifol J., Szabo P., Burri H.V., Canali C., Dufva M., Emneus J., Wolff A. (2015). Fabrication of scalable and structured tissue engineering scaffolds using water dissolvable sacrificial 3D printed moulds. Mater. Sci. Eng. C Mater. Biol. Appl..

[33] Dewey M.J., Johnson E.M., Weisgerber D.W., Wheeler M.B., Harley B.A.C. (2019). Shape-fitting collagen-PLA composite promotes osteogenic differentiation of porcine adipose stem cells. J. Mech. Behav. Biomed. Mater..

[34] Tyler B., Gullotti D., Mangraviti A., Utsuki T., Brem H. (2016). Polylactic acid (PLA) controlled delivery carriers for biomedical applications. Adv. Drug Deliv. Rev..

[35] Nguyen T.D., Kadri O.E., Sikavitsas V.I., Voronov R.S. (2019). Scaffolds with a High Surface Area-to-Volume Ratio and Cultured Under Fast Flow Perfusion Result in Optimal O2 Delivery to the Cells in Artificial Bone Tissues. Appl. Sci..

[36] Bruyas A., Lou F., Stahl A.M., Gardner M., Maloney W., Goodman S., Yang Y.P. (2018). Systematic characterization of 3D-printed PCL/beta-TCP scaffolds for biomedical devices and bone tissue engineering: Influence of composition and porosity. J. Mater. Res..

[37] Agrawal C.M., Kennedy M.E., Micallef D.M. (1994). The effects of ultrasound irradiation on a biodegradable 50–50% copolymer of polylactic and polyglycolic acids. J. Biomed. Mater. Res..

[38] Albrektsson T., Johansson C. (2001). Osteoinduction, osteoconduction and osseointegration. Eur. Spine J..

[39] Minardi S., Corradetti B., Taraballi F., Sandri M., Van Eps J., Cabrera F.J., Weiner B.K., Tampieri A., Tasciotti E. (2015). Evaluation of the osteoinductive potential of a bio-inspired scaffold mimicking the osteogenic niche for bone augmentation. Biomaterials.

[40] Vozzi G., Corallo C., Carta S., Fortina M., Gattazzo F., Galletti M., Giordano N. (2014). Collagen-gelatin-genipin-hydroxyapatite composite scaffolds colonized by human primary osteoblasts are suitable for bone tissue engineering applications: In vitro evidences. J. Biomed. Mater. Res. A.

[41] Zhang R., Ma P.X. (1999). Poly (α--hydroxyl acids)/hydroxyapatite porous composites for bone--tissue engineering. I. Preparation and morphology. J. Biomed. Mater. Res..

[42] Kothapalli C.R., Shaw M.T., Wei M. (2005). Biodegradable HA-PLA 3-D porous scaffolds: Effect of nano-sized filler content on scaffold properties. Acta Biomater..

[43] Midha S., Dalela M., Sybil D., Patra P., Mohanty S. (2019). Advances in three-dimensional bioprinting of bone: Progress and challenges. J. Tissue Eng. Regen. Med..

[44] Diment L.E., Thompson M.S., Bergmann J.H.M. (2017). Clinical efficacy and effectiveness of 3D printing: A systematic review. BMJ Open.

[45] Pang Y., Yao Y., Grottkau B. Rapid 3D Printing anatomically shaped bone scaffolds using novel molding and perfusion techniques. Proceedings of the TERMIS.

[46] Grottkau B.H., Zhi X., Pang Y. Rapid 3D printing of anatomically shaped bone scaffolds using novel molding and perfusion techniques. Proceedings of the AAOS.

[47] Guan J., Fujimoto K.L., Sacks M.S., Wagner W.R. (2005). Preparation and characterization of highly porous, biodegradable polyurethane scaffolds for soft tissue applications. Biomaterials.

[48] Pang Y., Yang J., Hui Z., Grottkau B.E. (2018). Robotic Patterning a Superhydrophobic Surface for Collective Cell Migration Screening. Tissue Eng. Part C Methods.

[49] Connerty H.V., Briggs A.R. (1966). Determination of serum calcium by means of orthocresolphthalein complexone. Am. J. Clin. Pathol..

[50] Olubamiji A.D., Izadifar Z., Si J.L., Cooper D.M., Eames B.F., Chen D.X. (2016). Modulating mechanical behaviour of 3D-printed cartilage-mimetic PCL scaffolds: Influence of molecular weight and pore geometry. Biofabrication.

